# The Impact of Topic Characteristics and Threat on Willingness to Engage with Wikipedia Articles: Insights from Laboratory Experiments

**DOI:** 10.3389/fpsyg.2017.01960

**Published:** 2017-11-07

**Authors:** Seren Yenikent, Peter Holtz, Joachim Kimmerle

**Affiliations:** ^1^Leibniz-Institut für Wissensmedien (IWM), Tübingen, Germany; ^2^Department of Psychology, University of Tübingen, Tübingen, Germany

**Keywords:** familiarity, controversiality, threat, mortality salience, uncertainty, Wikipedia

## Abstract

A growing body of research aims to identify the factors that motivate people to make contributions in Wikipedia. We conducted two laboratory experiments to investigate the connections between topic characteristics, perception of threat, and willingness to engage with Wikipedia articles. In Study 1 (*N* = 83), we examined how topic familiarity, topic controversiality, and mortality salience influenced participants’ willingness to engage with Wikipedia articles. We presented the introduction parts of 20 Wikipedia articles and asked participants to rate each article with respect to familiarity and controversiality. In addition, we experimentally manipulated participants’ level of mortality salience in terms of the amount of threat they experienced when reading the article. Participants also indicated their willingness to engage with a particular article. The results revealed that familiar and controversial topics increased the willingness to engage with Wikipedia articles. Although mortality salience increased accessibility of death-related thoughts, it did not result in any changes in people’s willingness to work with the articles. The aim of Study 2 (*N* = 90) was to replicate the effects of topic characteristics by following a similar procedure. We additionally manipulated uncertainty salience by assigning participants to three experimental conditions: uncertainty salience, certainty salience, and non-salience. As expected, familiar and controversial topics were of high interest in terms of willingness to contribute. However, the manipulation of uncertainty salience did not yield any significant results despite the emergence of negative emotional states. In sum, we demonstrated that topic characteristics were factors that substantially influenced people’s willingness to engage with Wikipedia articles whereas perceived threat was not.

## Introduction

Wikipedia is a free, collaboratively maintained online encyclopedia that contributors edit on a voluntary basis (Anthony et al., 2007). Wikipedia is the fifth most visited website in the world (Alexa, 2017). Following the successful principle of access to information by everybody (Ebner et al., 2006), it has developed into the largest and most prominent platform for presenting the knowledge of mankind (Yang and Lai, 2011). The English Wikipedia contains more than 5.4 million articles that have been written by 30 million registered and many unregistered users in the course of more than 900 million edits (Wikipedia, 2017). Being one of the most influential websites, it has triggered a large amount of research both about Wikipedia and using Wikipedia (Bar-Ilan and Aharony, 2014). These impressive numbers and achievements of Wikipedia have prompted many researchers to ask why people engage in such a voluntary-driven activity and what factors have an impact on their willingness to make active contributions to Wikipedia (Yang and Lai, 2011), instead of simply consuming information and avoiding active participation (Ebner and Holzinger, 2005). Several studies have attempted to identify what motivates contributions to Wikipedia (Rafaeli and Ariel, 2008; Xu and Li, 2015). Xu and Li (2015), for example, found that content contribution is often driven by extrinsic motivations, such as reciprocity and the need for self-development. Since Wikipedia is a collaborative project, a sense of community (Kuznetsov, 2006), a sense of belonging (Xu and Li, 2015), and collective agency (Jeong et al., 2017) are also crucial factors that have been reported as influencers of Wikipedia contributions in previous investigations. Other notable factors include cognitive benefits (Rafaeli et al., 2009), altruism (Kuznetsov, 2006), autonomy (Kuznetsov, 2006; Schroer and Hertel, 2009), fun, and ideology (Nov, 2007; see also Moskaliuk and Kimmerle, 2009). But in research on internet users’ willingness to engage with Wikipedia there are two other, largely unexplored, potentially relevant aspects: First, previous studies have neglected the impact of certain topic characteristics of the particular subject matter that people are potentially willing to engage with, that is, general properties of the Wikipedia articles themselves. Second, previous research has scarcely considered people’s moods, that is, particular emotional states that they experience in a given situation while using Wikipedia.

In the two experimental studies presented here, we measured the effects of those two kinds of motivational factors on people’s willingness to engage with particular Wikipedia articles. On the one hand, we set out to examine the role of the Wikipedia articles themselves. For this purpose, we varied the *topic characteristics* of the Wikipedia articles that people worked with. On the other hand, we examined people’s mood states while dealing with Wikipedia articles. Here, we manipulated the sense of *threat* that people were exposed to while they read certain Wikipedia articles as they were deciding whether they wanted to engage with them.

Particular characteristics of the topics that individuals choose are crucial elements that influence how they deal with the content. In general, it is well known that people attend to certain objects and activities that they care about and have interest in, and, accordingly, are willing to spend more time on (Renninger, 2000; Ainley et al., 2002). Cognitive elaboration of the content is enhanced or inhibited depending on the different aspects of individual interest regarding a particular topic (Ainley et al., 2002). This is also true on the internet: Internet users are more willing to engage with and spend more time dealing with online content that is interesting to them (Kubat and Tapia, 2007; Berger and Milkman, 2013). From this we argue that research on people’s willingness to engage with Wikipedia should take topic-related factors into account. Two characteristics that can be considered as appealing to internet users are topic familiarity and topic controversiality.

*Familiarity with a topic* depends on how often the topic comes up in a given cultural context (Gurkan, 2012). Personal and cultural relevance makes individuals easily create associations from real people, events, and places, which eventually leads to less cognitive load (Oller, 1995). Previous research has shown that participants performed better when they engaged with a culturally relevant topic (Lipson, 1983; Erten and Razi, 2009; Gurkan, 2012). Similar effects of topic familiarity were found as well for Wikipedia. In studies in which participants were instructed to evaluate the accuracy or credibility of Wikipedia articles, it was found that people focused more on the semantic features when they were familiar with the topics, whereas they paid more attention to surface characteristics such as length of the article when engaging with unfamiliar topics (Lucassen and Schraagen, 2011, 2012). In another study, West et al. (2012) examined the role of topic familiarity for Wikipedia authors’ involvement in edits. They found that authors made more and longer edits on the topics that they were familiar with. In the research presented here, we expected accordingly that people would be more willing to engage with Wikipedia articles on topics they were familiar with.

*Controversiality of a topic* is another purportedly important factor. Controversial topics are generally more attractive to people and increase the likelihood of conversation (Chen and Berger, 2013). Topics that create controversies and disagreements attract more attention in online communities as well. They lead to long conversations, discussions, and growth of communities (Sobkowicz and Sobkowicz, 2010; Chmiel et al., 2011; Buttliere and Buder, 2017). The controversiality of topics has also gained some attention in Wikipedia (Jirschitzka et al., 2017). For instance, the project of *Contropedia* aims to create a platform to identify societal controversies and to visualize knowledge about controversial topics in Wikipedia (Borra et al., 2014). Wilson and Likens (2015) conducted a study comparing the revision histories of some politically controversial and non-controversial Wikipedia articles and found that more edits were made and more words were changed on the controversial topics. Amongst the various types of controversial articles, topics of a socio-political nature get substantial coverage in Wikipedia (Yasseri et al., 2014). Based on this background, our studies here have also covered socio-politically controversial topics that create disputes in particular countries and which people are confronted with in everyday life. Considering the motivating effect of controversial topics, we expected that topic controversiality would lead to more willingness to engage with the articles.

When people use Wikipedia and decide whether or not to make contributions to a particular article, they are most likely influenced not only by topic characteristics of the articles they read, but also by the reactions they experience when reading about certain situations. Previous research has found, for example, that Wikipedia content covering threatening events (e.g., earthquakes and terror attacks) have an impact on their contributions to Wikipedia articles (Greving et al., 2017). We took up this line of research and aimed to examine the effect on people’s willingness to engage with Wikipedia articles which aroused in them a sense of threat. For this purpose, we have selected two key concepts from threat research (Jonas et al., 2014) and examined the impact of mortality salience and uncertainty salience in the Wikipedia context.

Threat research suggests that experiences which are discrepant from a person’s existing cognition or motivation may be perceived as a threat. If the discrepancy is manageable, people apply direct solutions. But if the current resources are not sufficient to dispel the source of the threat, a prolonged anxious and vigilant stage may occur and cause negative emotional states. In such situations, people try to reduce the unpleasant state (i.e., dissonance) and approach consonance through reduction of the discrepancy (Festinger, 1957; Cooper, 2011; Harmon-Jones and Harmon-Jones, 2012). The threatening information motivates them to conduct compensation behaviors to the extent that they want to maintain their existing beliefs (Gawronski, 2012). Two constructs that are studied in the theoretical framework of threat are mortality salience and uncertainty salience. These constructs explain people’s reactions in response to discrepant experiences by focusing on different aspects of threat. Mortality salience arises from instinctive concerns about death, whereas uncertainty is triggered by unpredictable situations in general (Van den Bos, 2004). In the following sections, we present mortality salience and uncertainty salience as examples of negative emotional states which cause a perception of threat that might have an impact on Wikipedia participation.

*Mortality salience* refers to people’s awareness that their own death is inevitable (Greenberg et al., 1994; Harmon-Jones et al., 1997). The mortality salience hypothesis posits that this awareness leads to high levels of terror and discomfort and influences people’s behaviors and preferences (Greenberg et al., 1986; Routledge et al., 2010). Individuals are inclined to adopt symbolic or actual compensations or defense behaviors in order to deal with the negative emotions that they experience in the state of mortality salience (Burke et al., 2010). Mortality salience can be induced by various cues in everyday situations. Internet users often come across death-related content which evokes their mortality awareness, and such content changes their online behavior patterns (Fransen et al., 2008; Chopik and Edelstein, 2014). We expect that when Wikipedia users encounter such content, in a Wikipedia article itself or elsewhere on the internet, it might then have an impact on their willingness to participate in Wikipedia as active contributors. The responses to threatening information are varied and might take several forms (Gawronski, 2012). Mortality awareness may lead people to feel that, considering the inevitable end, their existence and contributions to the world are meaningless and insignificant (Dechesne and Kruglanski, 2004). Thus, one might expect that this threat may undermine people’s willingness to engage in any activities. Such behavior as this would be consistent with previous research asserting that death cues have a hindering effect (e.g., Jonas et al., 2014). On the other hand, people who experience high levels of mortality awareness tend to produce something visible as a concrete testament which could endure beyond their lifetime (Solomon et al., 2004). Both of these reactions could be manifest in Wikipedia activities. Participants affected by mortality salience could be motivated to engage with Wikipedia articles as a means of participating in an activity that transcends the finality of their individual existence or they could be discouraged by their sense of finality from contributing to Wikipedia. In the current study, we approached the issue of these two possible behavioral reactions as an open research question.

Another aspect that we have included in the research presented here is the potential interaction between mortality salience and the topic characteristics of Wikipedia articles with respect to people’s willingness to engage with Wikipedia. In general, a feeling of threat changes individuals’ perceptions of their own environment and their needs in a given situation. In some cases, they are motivated to seek conformity with matching opinions in familiar contexts (Duckitt and Fisher, 2003). In other cases, induced death thoughts might lead people to defend their worldviews and make them willing to argue about issues that matter to them (Solomon et al., 2004). It is also reasonable to expect a need for safe and less chaotic environments under mortality salience (Duckitt and Sibley, 2010). Considering these connections between mortality salience and characteristics of the environment, we expected that mortality salience would interact with familiarity and/or controversiality of the Wikipedia articles to influence the participants’ willingness to engage. However, since the theoretical inferences about environmental characteristics were so varied, we did not make any formal hypotheses about interaction expectations. Instead, we examined the direction the interaction took as another open research question (e.g., whether people prefer to work with familiar or unfamiliar articles under mortality salience).

People are in need of cognitive clarity and consistency in their lives and employ cognitive mechanisms and behaviors to restore and retain a certain amount of clarity (Hogg, 2000; Jonas et al., 2014). Threats that upset the certainty balance often cause defensive behaviors. *Uncertainty management* is considered to be one of the most prominent factors influencing people’s behavior, as uncertainty is confronted almost on a daily basis in the form of rapid changes and unpredictability in personal, occupational, and political worlds (Van den Bos, 2001; Van den Bos et al., 2005). In ambiguous situations, people desire to find answers to avoid or diminish unpredictability (Kruglanski and Webster, 1996). Their cognitive system starts to work on generating hypotheses to resolve the state of uncertainty (Kruglanski, 1990). Dealing with the uncertainty of the environment is an important element of human experience and behavior in many contexts (Hogg, 2007), including the internet. The uncertainty of online environments affects people’s activities (Lim et al., 2004; Pavlou et al., 2006). This is likely to be also true of Wikipedia, as some Wikipedia articles deal with phenomena that are themselves uncertain or unpredictable to a certain degree (Vincze, 2013). For instance, Wikipedia content that contains cues of discrepancy and tentativeness significantly influence editors’ activities (Yenikent et al., 2017, Unpublished). This makes it relevant to investigate the effects of uncertainty within the Wikipedia context as well.

Our assumption was that people who are preoccupied with a focus on uncertainty would be less willing to work with new information provided, such as information in Wikipedia articles. Furthermore, familiar and non-controversial topics should be more appealing to participants in a state of uncertainty, as such topics would allow them to reduce the effort required for processing new and complex information (Webster and Kruglanski, 1994), and would allow them to feel more comfortable in a predictable environment (Duckitt and Sibley, 2010).

Our goal was to extend the theoretical considerations and previous findings with regard to topic characteristics and perception of threat to the Wikipedia setting, to gain insights about the various ways these factors have an impact on people’s willingness to engage with Wikipedia. In particular, we aimed to understand whether familiarity with and controversiality of a topic influenced people’s willingness to engage with Wikipedia articles. A further aim was to examine whether inducing mortality and uncertainty saliences would influence people’s willingness in this setting. We conducted two experimental studies to achieve our research goals. Study 1 focused on analyzing participants’ willingness to engage with Wikipedia articles depending on their familiarity with the topic, the controversiality of the topic, and induction of mortality salience. In Study 2, we also took topic familiarity and controversiality into account, but manipulated uncertainty salience and measured their effects on participants’ willingness to engage with Wikipedia articles. Ethical approval was obtained for both studies from the institutional board of ethics (approval number: LEK 2016/025).

## Study 1

In Study 1, we examined the effects of topic familiarity, topic controversiality, and mortality salience on willingness to engage with Wikipedia topics. As outlined above, previous research suggests that people are particularly interested in familiar as well as in controversial topics. On the basis of these considerations we stated the following hypotheses:

H1a: Participants are more willing to engage with familiar than with unfamiliar Wikipedia articles.
H1b: Participants are more willing to engage with controversial than with non-controversial Wikipedia articles.

As explained above, one may assume that mortality salience would reduce people’s willingness to engage with Wikipedia articles, since mortality awareness could lead people to feel that their contributions would be meaningless. One may also assume that people who experience high levels of mortality awareness could have the opposite reaction and would particularly want to produce visible testaments, increasing their willingness to engage with Wikipedia articles. Accordingly, we stated the following as an open research question:

RQ1: It is an open research question whether participants who are exposed to mortality salience are more or less willing to engage with Wikipedia articles than participants who are not exposed to mortality salience.

Based on our previous theoretical considerations about mortality salience, we assumed that mortality salience would interact with each of the topic characteristics. However, as there are conflicting theoretical inferences, we did not make any particular, formal hypotheses, but formulated instead open research questions:

RQ2: There are interaction effects between mortality salience and each of the perceived topic characteristics.
RQ2a: It is an open research question whether participants who are exposed to mortality salience are more willing to engage with familiar or with unfamiliar Wikipedia topics.
RQ2b: It is an open research question whether participants who are exposed to mortality salience are more willing to engage with controversial or with non-controversial Wikipedia topics.

### Method

#### Participants

Ninety-seven participants volunteered to take part in the experiment. In order to make sure that participants had similar experiences with the topics (see below), we had to exclude 14 participants who reported a country of origin other than Germany, a mother tongue other than German, or another language that they spoke at home and/or with their parents (especially those who reported Turkish language and origins, as we used Germany and Turkey topics in contrast. Please see below). Therefore, the final analyses were run based on the reports from 83 participants, 53 of which were females. The participants’ ages ranged between 19 and 64 years old (*M* = 26.4, *SD* = 8.5). Sixty-one participants reported that they read Wikipedia articles at least once a week. They indicated an average reading time of *M* = 1.57 h per week (*SD* = 1.11). Only two participants claimed to be Wikipedia authors.

#### Instruments and Material

##### Wikipedia articles

We used 20 different topics from the German-language version of Wikipedia. The introduction parts of the articles were displayed as screenshots from the actual Wikipedia pages. The length of these introduction texts varied between 37 and 172 words. The topics were selected based on an assumed variance regarding familiarity and controversiality to fit four categories: familiar/controversial, familiar/non-controversial, unfamiliar/controversial, and unfamiliar/non-controversial. Since, we were working with a German sample, familiar topics addressed German culture and society. Articles which had to do with Turkey were selected as unfamiliar topics. In terms of controversiality, topics ranged from hotly debated societal issues and highly disputed public figures on the one hand, to topics such as geographical and undisputed historical articles on the other, which did not have any relation to debated issues in these two countries (see **Table 1**). The alignment between pre-defined categories and participants’ perception of topic characteristics was examined via manipulation check analysis (see below).

**Table 1:** Wikipedia topics in Study 1.

	Familiar	Unfamiliar
Controversial	Refugee crisis in Germany 2015	Corruption scandal in Turkey 2013
	Social focal point	Alevism
	Thilo Sarrazin	Abdullah Ocalan
	Homosexuality in Germany	Homosexuality in Turkey
	Speed limits in Germany	Ergenekon
Non-controversial	Mainz	Yazilikaya
	Bavarian Forest	Cappadocia
	German navy history	Seljuq dynasty
	Elbe	Pontic Mountains
	Gerd Mueller	Baris Manco


*This table provides English translations of the German Wikipedia page titles.*

##### Experimental manipulation

Following methods of similar studies (e.g., Rosenblatt et al., 1989), mortality was induced by providing the treatment group with the following instructions: “Please think of your own death and what will happen to you physically when you die. Write down your emotions about it.” The control group, in contrast, received the following request: “Please think of a situation in which you felt the joy of life. Write down your emotions about it.” We analyzed the written material in order to check whether the mortality induction yielded differences in emotional states (see below).

##### Questionnaires

*Perceived topic characteristics:* This scale consisted of two items: Participants rated each of the 20 articles in terms of how familiar they were with the topic and in terms of how controversial they thought the topic was on a 7-point Likert scale ranging from 1 (*not at all*) to 7 (*very much*).

*Willingness to engage with Wikipedia articles:* This scale was developed to measure participants’ willingness to engage with each Wikipedia topic. It consisted of four items to be responded to on 7-point Likert scales ranging from 1 (*not at all*) to 7 (*very much*). We asked for the participants’ willingness to engage with each topic in terms of (1) reading more about it on Wikipedia, (2) editing the Wikipedia article, (3) joining the talk pages of the articles, and (4) delving into the topic outside Wikipedia. The internal consistency of this scale was excellent (Cronbach’s α = 0.970).

#### Procedure

Participants were recruited via an online participant pool and invited to the laboratory. They completed the survey on a computer provided to them in a cubicle. After reading the instructions and giving informed consent, participants were asked to indicate their demographics and information about their Wikipedia usage. In the first part of the experiment, participants were randomly shown the Wikipedia topics one at a time and instructed to rate their familiarity with each topic and its controversiality. After this step, they were randomly assigned to the experimental and control conditions and asked to carry out the given task. Then the same topics were once again shown to the participants one after the other and they were asked to fill out the willingness scale for each topic. The experiment ended with a debriefing page, where the purpose of the study was explained.

### Results and Discussion

#### Manipulation Checks

Before the analyses, we conducted two manipulation checks. First of all, we examined the alignments between the pre-defined topic categories and the participants’ ratings of Wikipedia articles. Familiarity ratings for the familiar articles (*M* = 3.241, *SD* = 0.913) significantly differed from the ratings of the unfamiliar articles (*M* = 1.665, *SD* = 0.682), *t*(82) = 20.030, *p* < 0.001. Also, controversial articles (*M* = 5.238, *SD* = 0.885) and non-controversial articles (*M* = 1.839, *SD* = 0.638) differed significantly from each other in terms of controversiality ratings, *t*(82) = 35.822, *p* < 0.001. These findings allowed us to apply these categories as two factors in further analyses.

We also took into account whether the mortality manipulation elicited different emotional states across conditions. We implemented a sentiment analysis on the text written by the participants during the experimental task. The texts were analyzed with the LIWC 2001 German dictionary (Linguistic Inquiry and Word Count; Wolf et al., 2008), a software that analyzes the relative frequencies of words with regard to linguistic and psychological processes. We found a significant difference in the frequency of death-related words written by the mortality group (*M* = 5.749, *SD* = 4.248) and the control group (*M* = 0.963, *SD* = 0.608), *t*(81) = 8.333, *p* < 0.001. Participants in the mortality group also expressed significantly more negative emotions (*M* = 7.669, *SD* = 11.349) than participants in the control group (*M* = 1.220, *SD* = 2.281), *t*(81) = 3.527, *p* = 0.024. Evidently, mortality induction resulted in a negative emotional state in the manipulation group.

#### Main Analysis

In order to test the hypotheses and answer the research questions we ran a 2 (familiar vs. unfamiliar) × 2 (controversial vs. non-controversial) × 2 (mortality salience vs. control) mixed ANOVA on willingness to engage with Wikipedia articles. Familiarity and controversiality were within-participants factors, whereas mortality was a between-participant factor.

**FIGURE 1:**
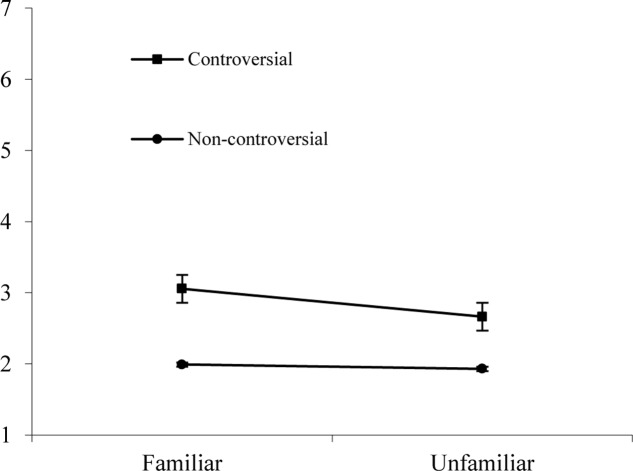
Interaction effect of familiarity and controversiality on willingness to engage with Wikipedia articles in Study 1.

The analysis showed a significant main effect of familiarity on willingness (H1a), *F*(1,81) = 11.704, *p* < 0.001, $\eta_{p}^{2}$ = 0.126. Participants were more willing to engage with familiar (*M* = 2.522, *SD* = 1.493) than with unfamiliar topics (*M* = 2.294, *SD* = 1.392). We also found a main effect for controversiality (H1b), *F*(1,81) = 71.245, *p* < 0.001, $\eta_{p}^{2}$ = 0.468. Participants were more willing to engage with controversial articles (*M* = 2.855, *SD* = 1.529) than with non-controversial articles (*M* = 1.960, *SD* = 1.205). Familiarity and controversiality also interacted with each other, *F*(1,81) = 9.844, *p* < 0.01, $\eta_{p}^{2}$ = 0.108, indicating that people were most willing to engage with articles that were familiar and controversial (*M* = 3.055, *SD* = 1.217). These effects are shown in **Figure 1**.

In contrast to our expectations, there was no main effect of mortality salience on willingness (RQ1), *F*(1,81) = 0.676, *p* = 0.413. Mortality salience did also not interact with familiarity (RQ2), *F*(1,81) = 3.003, *p* = 0.087, or controversiality, *F*(1,81) = 0.140, *p* = 0.710.

To sum up, we found supporting evidence for impact of topic characteristics; as expected, participants preferred to work on topics that were familiar to them and that they perceived to be controversial. Participants were particularly willing to engage with articles that were familiar and controversial. Regarding RQ1, the induction of mortality salience did not have any significant impact on participants’ willingness to engage with a topic. This result is not congruent with the claims of the Terror Management Theory (Greenberg et al., 1986) that suggests that mortality salience could alter people’s behaviors and preferences. Regarding RQ2, we did not find any supporting evidence, as mortality salience did not interact with either familiarity or controversiality.

## Study 2

In Study 2, we examined the influence of topic familiarity, topic controversiality, and uncertainty salience on willingness to engage with Wikipedia articles. First of all, we aimed to replicate the findings from Study 1. Thus, we once again stated the following hypotheses:

H1a: Participants are more willing to engage with familiar than with unfamiliar Wikipedia articles.
H1b: Participants are more willing to engage with controversial than with non-controversial Wikipedia articles.

As outlined above, there are theoretically and empirically justified reasons to assume that the induction of uncertainty undermines people’s willingness to engage with Wikipedia articles. Therefore, we expected the following:

H2: Participants who are exposed to uncertainty salience are less willing to engage with Wikipedia articles than participants who are not.

Finally, the following hypotheses address the considerations stated above concerning uncertainty salience and its effect on the preference for familiar and non-controversial topics:

H3: There are interaction effects between uncertainty salience and each of the perceived topic characteristics.
H3a: Participants who are exposed to uncertainty salience are more willing to engage with familiar Wikipedia topics than participants who are not exposed to uncertainty salience.
H3b: Participants who are exposed to uncertainty salience are more willing to engage with non-controversial Wikipedia topics than participants who are not exposed to uncertainty salience.

### Method

#### Participants

One hundred participants took part in the second laboratory experiment. We had to exclude 10 participants who indicated non-German origins, a mother tongue other than German, or another language that they spoke at home and/or with their parents (including Turkish origins and language) from the original data. The remaining 90 participants were included in the final analysis. Participants were between 18 and 60 years old (*M* = 23.87; *SD* = 6.32); 59 of the 90 participants were female. Sixty-eight participants indicated they read Wikipedia articles at least once a week, with an average reading time of *M* = 1.89 h (*SD* = 3.90). Two participants were active Wikipedia authors.

#### Instruments and Material

##### Wikipedia articles

In Study 2, we presented 12 Wikipedia articles, 11 articles selected from the first study and one new article (see **Table 2**). The aim of this selection was to apply a set of topics that were rated as highly familiar, controversial, unfamiliar, and non-controversial, respectively. Thus, we removed nine topics that were rated as moderately familiar and controversial in Study 1. The introduction parts of the articles were again presented as screenshots. The length of these introduction texts varied between 45 and 142 words. We implemented a manipulation check comparing participants’ familiarity and controversiality ratings with our categories (see below).

**Table 2:** Wikipedia topics in Study 2.

	Familiar	Unfamiliar
Controversial	Refugee crisis in Germany 2015	Corruption scandal in Turkey 2013
	Thilo Sarrazin	Abdullah Ocalan
	Homosexuality in Germany	Homosexuality in Turkey
Non-controversial	Mainz	Yazilikaya
	Feldberg^∗^	Cappadocia
	Elbe	Pontic Mountains


*This table provides English translations of the German Wikipedia page titles. ^∗^ Newly added topic.*

##### Experimental manipulation

On the basis of earlier research (Van den Bos, 2001), we asked participants to think of one of the following situations and write down their emotions caused by those thoughts: (1) an *unresolved personal dilemma* (uncertainty salience condition), (2) an *easy personal decision* (certainty salience condition), or (3) *watching television* (non-salience condition). The texts that participants wrote down during the manipulation task were further analyzed to compare their emotional states across conditions (see below).

##### Questionnaires

*Perceived topic characteristics:* The scale had the same purpose as in Study 1 and included the same items. Participants rated each of the 12 articles in terms of how familiar they were with the topic and in terms of how controversial they thought the topic was on a 7-point Likert scale ranging from 1 (*not at all*) to 7 (*very much*).

*Willingness to engage with Wikipedia articles:* We used the scale from Study 1 but rephrased the third item in favor of broader wording (now asking for the willingness to collaborate with other people on the topic). Participants responded on 7-point Likert scales ranging from 1 (*not at all*) to 7 (*very much*). Internal consistency was again excellent (Cronbach’s α = 0.949).

#### Procedure

As in the first study, this study was implemented as a laboratory experiment. Participants were recruited from an online participant pool and invited to the laboratory to fill out a survey on a computer screen in a cubicle. The experiment started with an instructions page, also including the consent form, followed by inquiry into their demographics and Wikipedia usage. Participants were randomly assigned to three experimental conditions and then carried out the task. After the manipulation, they were shown the Wikipedia topics one at a time and asked to rate the perceived topic characteristics and willingness to engage for each topic. After completing the study, participants were debriefed.

### Results and Discussion

#### Manipulation Checks

Again, we conducted manipulation checks before the main analyses. First, we ensured the validity of the pre-defined topic categories regarding familiarity and controversiality. Familiarity ratings for familiar articles (*M* = 3.218, *SD* = 0.917) differed significantly from the ratings of unfamiliar articles (*M* = 1.663, *SD* = 0.642), *t*(89) = 19.877, *p* < 0.001. Likewise, controversial articles (*M* = 5.07, *SD* = 0.987) differed significantly from non-controversial articles (*M* = 1.498, *SD* = 0.613) regarding participants’ controversiality ratings, *t*(89) = 30.684, *p* < 0.001. These results enabled us to use these categories as two experimental factors.

The LIWC 2001 German dictionary (Wolf et al., 2008) was used to analyze the written material obtained through the experimental task, in order to assess whether the induction of uncertainty yielded differences in participants’ emotional states. An ANOVA yielded significant variations in negative emotions among conditions, *F*(2,87) = 4.901, *p* = 0.010. A Tukey *post hoc* test revealed that the uncertainty group (*M* = 4.131, *SD* = 4.351) had significantly higher negative emotion scores than the certainty group (*M* = 1.569, *SD* = 2.555, *p* = 0.009) and non-salience group (*M* = 1.925, *SD* = 3.077, *p* = 0.048). There was no difference between the certainty and non-salience groups (*p* = 0.901).

#### Main Analysis

For testing the hypotheses, we conducted a 2 (familiar vs. unfamiliar) × 2 (controversial vs. non-controversial) × 3 (uncertainty salience vs. certainty salience vs. non-salience) mixed ANOVA, where familiarity and controversiality were within factors and uncertainty was a between factor. We found a main effect of controversiality (H1b), *F*(1,87) = 86.999, *p* < 0.001, $\eta_{p}^{2}$ = 0.500. Participants were more willing to engage with controversial articles (*M* = 3.335, *SD* = 1.433) than non-controversial articles (*M* = 2.123, *SD* = 1.144). Familiarity (H1a), *F*(1,87) = 0.409, *p* = 0.524, and uncertainty salience (H2), *F*(2,87) = 1.949, *p* = 0.149 did not show main effects on willingness.

**FIGURE 2:**
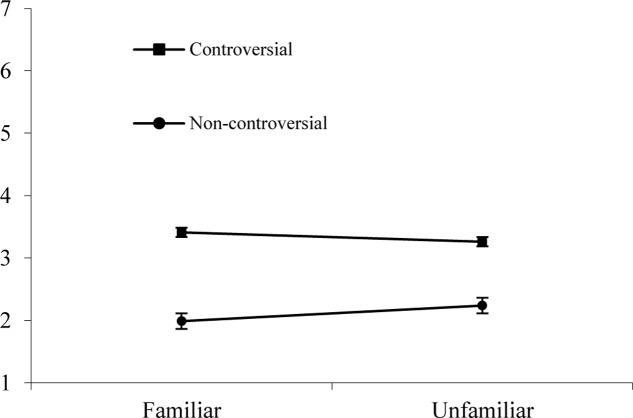
Interaction effect of familiarity and controversiality on willingness to engage with Wikipedia articles in Study 2.

Contrary to our expectations, the analysis did neither show an interaction effect of familiarity and uncertainty salience (H3), *F*(2,87) = 1.898, *p* = 0.156, nor of controversiality and uncertainty salience, *F*(2,87) = 0.681, *p* = 0.509. However, we found an interaction effect between familiarity and controversiality, *F*(2,87) = 8.670, *p* < 0.01, $\eta_{p}^{2}$ = 0.091 (see **Figure 2**): Participants were most willing to engage with articles that were familiar and controversial (*M* = 3.412, *SD* = 1.071).

In conclusion, with Study 2 we replicated the significant influence of topic controversiality on willingness to engage with Wikipedia articles. Participants showed more willingness to contribute to controversial articles than they did for non-controversial articles; this was not the case for familiar and unfamiliar topics although again participants had the highest willingness scores for the familiar/controversial topic type. Contrary to our expectations, uncertainty induction did not affect participants’ willingness to engage with Wikipedia articles. Finally, topic characteristics did not show any interaction effects with uncertainty salience.

## General Discussion

As a social software site relying upon a completely open editing system (Lih, 2004), Wikipedia is a widely used tool in everyday online learning. The principle of universal access makes information available for everybody (Ebner et al., 2008). Features such as article talk pages allow editors to debate about the topics, and large numbers of authors with diverse backgrounds contribute to high quality knowledge construction processes (Kittur et al., 2007; Kittur and Kraut, 2008; Oeberst et al., 2014). Its important role in knowledge construction processes has led to great interest in research to identify the factors influencing contributions to Wikipedia. In our laboratory studies, we empirically examined the effects of two factors, topic characteristics and threat perception, on the willingness to engage with Wikipedia. The first important finding was the substantial impact of topic familiarity and topic controversiality on willingness to engage with Wikipedia topics. This suggests that people’s motivation to work on Wikipedia articles depends on their perception of the familiarity and controversiality of the content. Moreover, articles that were both familiar and controversial were the most engaging; it is likely that by engaging with culturally relevant (Carrell, 1987) and socio-politically controversial (Yasseri et al., 2014) topics, people get the chance to express their opinions on hotly debated issues that are relevant to them.

Using considerations from threat theories as a basis, we also examined the role that feelings of threat play in a given Wikipedia context. We first investigated the manipulation of mortality thoughts by taking on this issue as an open research question. Contrary to our expectations, mortality salience did not significantly affect people’s willingness to work on Wikipedia articles. This result does not support the mortality salience literature that suggests that people get actively involved in meaning-making processes to maintain a sense of self-esteem after mortality induction (Arndt et al., 2004). Perception of significant threats to survival provokes negative associations related to death and people adopt defense behavior to reduce death-thought accessibility. Our participants did in fact report more death-related words and negative emotions after the mortality manipulation. However, this negative mood state does not seem to have spilled over to their engagement to Wikipedia articles. In the dual-process model of the Terror Management Theory, Pyszczynski et al. (1999) state that death-related thoughts lead to conscious proximal defenses, whereby people first try to rationally deny their vulnerability to death, and unconscious distal defenses, whereby people take indirect symbolic defense behaviors (Greenberg et al., 2000). While we found that conscious death-related thoughts were primed in participants’ minds, we did not find evidence that this motivated them to contribute more to Wikipedia in order to reduce the death-thought accessibility in the next phase. Distractions or subtle death cues might have helped participants to push the death-thought accessibility out of consciousness (Greenberg et al., 1994) which might have in turn activated indirect distal defenses in the form of a motivated or inhibited Wikipedia participation. Based upon our findings, it is plausible to state that people’s emotional states and death-thought accessibility may be influenced by the death inductions; yet, this effect might not be sufficient to change their willingness to engage with Wikipedia. More research is needed to understand whether distal defenses emerge in Wikipedia users’ contributions in the long run.

Following the research line which suggests that other existential concerns, such as certainty needs, are as important as mortality awareness (Koole et al., 2004), we manipulated in Study 2 uncertainty salience as another feature of threat perception. Contrary to our hypothesis and literature, uncertainty salience did not have any effect on participants’ willingness to engage with Wikipedia. This result does not corroborate the role of situational certainty in knowledge construction (Kruglanski, 1990) or previous literature positing effects of uncertainty induction on behavioral outcomes (Van den Bos et al., 2005). We instructed participants to conjure up personal experiences since self-related uncertainty entails negative mood and striving to reduce the discomfort of uncertainty feelings (Hogg et al., 2007). As expected, participants reported negative emotions after the induction of uncertainty; however, their mood did not have effects on their Wikipedia contributions. The concept of uncertainty has various aspects that include personality dispositions (Neuberg and Newson, 1993), contextual factors (Kruglanski et al., 2006), and social, cognitive, and perceptual challenges (Hogg, 2007). Hence, this complex nature might have inhibited the direct effect of uncertainty manipulation on the outcome behavior.

Previous research on the phenomenon of threat perception allowed us to construct interaction hypotheses between topic characteristics on the one hand and mortality and uncertainty saliences on the other. Both mortality awareness and certainty management research propose that cues of death and uncertainty cause reactive behaviors (Jonas et al., 2014). From this we assumed that people would demonstrate different topic preferences in mortality- and uncertainty-salient conditions. However, in both studies we found no empirical support for our interaction hypotheses. Non-significant effects of mortality salience and uncertainty salience on willingness to engage with Wikipedia, could indicate that the manipulation variables might not have been strong enough to interact with topic characteristics. It could be that the mortality and uncertainty manipulations might have been insufficiently strong to alter participants’ perceptions of their own environments.

Our findings have implications for optimizing search queries. Today’s technology allows tracking web search behaviors on a large scale (White et al., 2009). Different types of familiar and/or controversial Wikipedia topics could be suggested to users depending upon their web search and/or Wikipedia queries. In a user-generated platform like Wikipedia (di Sciascio et al., 2017), tailoring queries based on users’ interests could significantly contribute to the knowledge quality.

One limitation in our study is related to the scale used to measure participants’ willingness to engage with articles. Even though the experiments were clearly conducted in a Wikipedia setting, we had one item that measured general interest in the topics outside the Wikipedia context. This issue may make it difficult to draw specific inferences in terms of willingness to work with the topics in Wikipedia; the results might instead indicate a broad, general interest in the topics. Future studies focusing on the reasons for people’s willingness to engage with Wikipedia could be improved by refinement of the willingness scale. Our studies are also limited in terms of overlooking the potential interactions between particular topics and participants’ personal opinions on those topics. Previous investigations showed that people could be affected by their own biases while working with Wikipedia articles (Beck et al., 2017; Oeberst et al., 2017). An opinion bias might have played a role in participants’ motivation to engage with articles in our studies as well. Future research should take individuals’ opinions on topics into account and examine whether these opinions have an impact on engagement with the respective Wikipedia articles.

In spite of these limitations, our studies could be a good starting point for further investigations that could look into actual Wikipedia settings on the internet, especially considering the fact that laboratory experiments provide solid results for causal inferences (Falk and Heckman, 2009). Future research could address particular types of topic characteristics, in terms of identifying particular content that would predict editors’ activities. Another interesting direction for future investigation would be to scrutinize the relationship between Wikipedia authors’ mood states and threat perceptions, and examine whether this relationship influences their Wikipedia participation.

## Author Contributions

SY, PH, and JK made substantial contributions to the conception and design of the work; SY and PH were involved in the analysis and interpretation of data for the work. SY and JK drafted the work; PH revised it critically for important intellectual content. SY, PH, and JK approved the final version to be published. SY, PH, and JK agree to be accountable for all aspects of the work in ensuring that questions related to the accuracy or integrity of any part of the work are appropriately investigated and resolved.

## Conflict of Interest Statement

The authors declare that the research was conducted in the absence of any commercial or financial relationships that could be construed as a potential conflict of interest.
